# Effect of Different Exercise Interventions on Grip Strength, Knee Extensor Strength, Appendicular Skeletal Muscle Index, and Skeletal Muscle Index Strength in Patients with Sarcopenia: A Meta-Analysis of Randomized Controlled Trials

**DOI:** 10.3390/diseases12040071

**Published:** 2024-04-02

**Authors:** Xinxiang Wang, Lijuan Wang, Yu Wu, Ming Cai, Liyan Wang

**Affiliations:** 1 College of Rehabilitation, Shanghai University of Medicine & Health Sciences, Shanghai 200237, China; tyky@synu.edu.cn (X.W.);; 2 College of Sports Science, Shenyang Normal University, Shenyang 110034, China

**Keywords:** sarcopenia, grip strength, knee extensor strength, appendicular skeletal muscle index, skeletal muscle index

## Abstract

Sarcopenia is a systemic skeletal muscle disease that is more prevalent in older adults. The role of exercise in improving the disease has been demonstrated. However, due to the variety of exercise modalities, it is not clear what type of exercise provides the best benefit. The aim of this meta-analysis was to analyze the effects of different exercise modalities on grip strength, appendicular skeletal muscle index, skeletal muscle index, and knee extensor strength in elderly patients with sarcopenia. The protocol for this evaluation was registered on the PROSPERO website and the databases PubMed, WOS, Cochrane Library, and Embase were searched. Thirteen studies were included in the analysis. The results showed that exercise interventions had positive effects on grip strength and knee extension muscle strength, with resistance training being the most effective. There was no significant improvement in appendicular skeletal muscle index or skeletal muscle index. This study still has limitations. For example, age group and exercise duration were not considered. Future studies should further explore benefits in age groups as well as other relevant outcome indicators.

## 1. Introduction

Sarcopenia is a hallmark of excessive aging and is also widely recognized as a progressive and systemic skeletal muscle disease involving a rapid decline in muscle mass, muscle strength, and physical functioning [1]. This condition is notably prevalent among older adults. Still, it is customary to begin experiencing a loss of muscle mass after 30 years, with an average loss of 5–10% per decade [2], and muscle strength declines at a more dramatic rate than muscle mass [3]. This, in turn, speeds up the decline in sports performance and skeletal muscle mass. In addition, sarcopenia has been shown to be associated with many adverse health outcomes, such as falls, functional impairment, poor quality of life, and even mortality [1,4,5]. Despite this, the effective management and clinical treatment of the disease are further hindered by the current inconsistency in the definition of sarcopenia and the lack of consensus on diagnostic criteria [3].

At present, while there are no exact and effective pharmacological treatments in older adults with sarcopenia, the positive health benefits of exercise as a non-pharmacological intervention have been widely acknowledged by scholars, with strong evidence supporting its effectiveness [6,7]. Numerous studies have found that exercise produces significant physiological and health benefits, playing a key role in preventing or delaying the progression of sarcopenia [8,9,10]. Previous research has demonstrated that various forms of exercise can have a beneficial effect on the management of sarcopenia in older adults [11,12]. These exercise modalities, such as resistance training, whole-body vibration training, and aerobic training, are commonly used in clinical practice. Resistance training is considered the most commonly used and effective exercise modality in clinical practice [8,13,14] and is the only intervention consistently shown to improve outcomes for older adults with sarcopenia [15,16,17].

Several systematic reviews and meta-analyses have provided evidence showing the efficacy of exercise on muscle strength and muscle mass in older adults with sarcopenia. A systematic review and meta-analysis showed that exercise programs had a positive effect on muscle strength [8]. As far as muscle strength is concerned, the authors only analyzed grip strength (GS), chair standing time, physical performance (gait speed), and the timed up and go. In a review by Vlietstra L, the results showed that exercise interventions significantly improved muscle strength and muscle mass [10]. The indicators of concern in this author’s study included knee extensor strength (KES), timed up and go, appendicular muscle mass, and leg muscle mass. However, regarding the diagnosis and assessment of sarcopenia, according to the International Working Group’s diagnostic criteria for sarcopenia, it was emphasized that emphasis should be placed on the assessment of patients’ muscle strength and muscle mass [18], for example, exploring the benefits of GS, knee extensor strength, appendicular skeletal muscle index (ASMI), and gait speed. At the same time, consensus on the diagnosis of sarcopenia, especially with regard to muscle mass, should suggest an assessment that links ASMI or skeletal muscle index (SMI) [19]. Therefore, it may be crucial to prevent and delay the decline in muscle strength and muscle mass in older adults with sarcopenia. In this regard, we selected the main indicators of GS, KES, ASMI, and SMI to assess muscle strength and muscle mass in older adults with sarcopenia. The aim was to conduct a meta-analysis of randomized controlled trials to better understand the role of exercise in the treatment of sarcopenia and to contribute to the development of effective exercise interventions for this population.

## 2. Materials and Methods

### 2.1. Information Sources and Search

**Figure 1:**
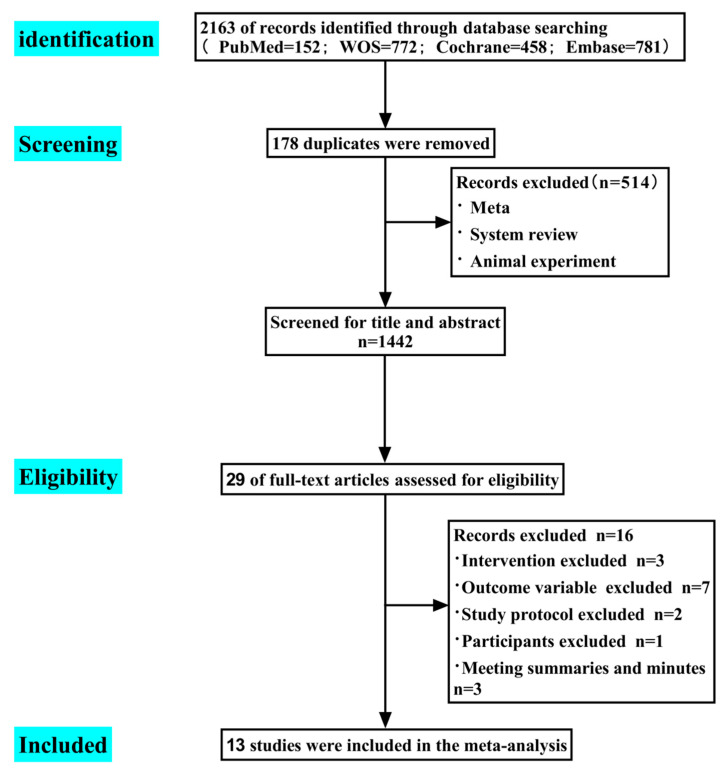
Flowchart of the literature search and literature inclusion.

The meta-scheme of this study has been registered in the PROSPERO International Prospective Systematic Review Registry (CRD: 42023437768). All randomized controlled trial articles from 1997 to January 2023 were retrieved according to the Preferred Reporting Items for Systematic Reviews and Meta-Analyses (PRISMA) in the following electronic databases for Completed: PubMed, Web of Science Core Collection, Cochrane Library, and Embase [20]. The article selection process used a PRISMA flowchart to record the number of articles selected or excluded at each step (Figure 1). The complete search strategy is presented in Supplementary Materials S1. The screening of abstracts and titles was performed independently by two reviewers who assessed the quality of the articles, and any disagreements were resolved by consensus. If differences persisted, they were discussed and fixed with a third reviewer.

### 2.2. Eligibility Criteria

#### 2.2.1. Inclusion

Eligibility criteria for the original studies were assessed according to the PICOS principles. Articles using the following conditions were considered to meet the inclusion criteria: (1) the language of the article was English, (2) it was a randomized controlled trial, (3) the age of the subjects was 60 years or older, (4) the outcome metrics were reported in at least any of the primary or secondary outcomes of the original article, (5) there was a diagnosis of sarcopenia according to any of the definitions or decrease in muscle mass, muscle strength, and physical function decline by any definition, (6) there were various modalities of exercise intervention.

#### 2.2.2. Exclusion

The exclusion criteria were as follows: (1) age less than 60 years, (2) animal experiments, (3) non-randomized controlled trial articles, (4) duplicate reports, (5) presence of other diseases, (6) conference abstracts, newsletters, and announcements.

### 2.3. Data Extraction

The article selection process was documented using the PRISMA 2020 flow diagram template to record studies selected or excluded at each step. One author (X.W.) performed data extraction independently using EndnoteX9.1 software for articles that met the inclusion criteria and this were then checked by a second author (Y.W.). To ensure the inclusion of original studies that met the inclusion criteria, two researchers (X.W. and Y.W) independently screened the articles based on their titles and abstracts using Endnote X9.1 software. Any article that may have met the criteria was to be screened by two researchers (X.W. and Y.W.) reading the full article. Disagreements arising during the screening process were to be discussed and resolved with a third researcher (L.W.). Finally, forest plots, funnel plots, sensitivity analyses, and clipping methods were jointly completed through Review Manager 5.4 and Stata 14 software.

In this paper, we extracted the following information from the included articles: demographical information (year, author, sample size, setting, country, age), measurement tool, type of device, GS, KES, muscle mass, SMI, and ASMI. In addition, we extracted the control group from the raw data, baseline, and post-intervention means and standard deviations of the experimental group.

### 2.4. Risk of Bias Assessment

The Cochrane risk-of-bias assessment tool was developed on the recommendations of the Cochrane Handbook. It was completed independently by two authors using the Cochrane Risk of Bias 2 (RoB 2) tool to assess the risk of bias. It included studies based on seven dimensions of sources of bias and six significant aspects: selection bias, performance bias, detection bias, attrition bias, reporting bias, and other biases. Each entry was categorized into three types of bias: low risk of bias, high risk of bias, and unclear risk of bias. 

**Figure 2:**
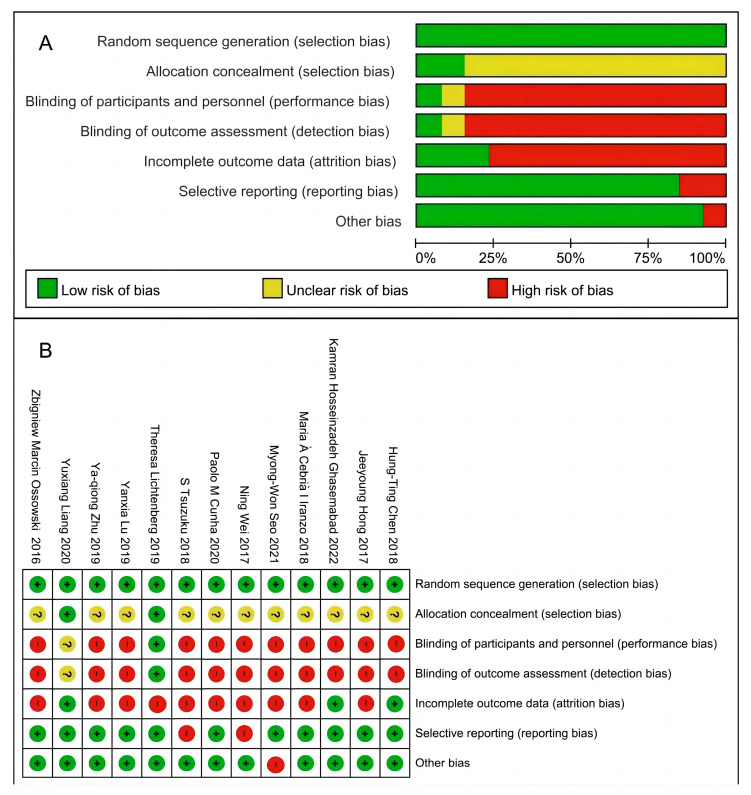
Assessment of risk of bias based on the Cochrane risk-of-bias tool. (**A**) Risk of bias summary; (**B**) Risk of bias graph.

The final combined results are shown in Figure 2. 84.6%, 7.7%, and 7.7% of studies had unclear risk of bias (allocation concealment, blinding of participants and personnel, and blinding of outcome assessment); 84.6% of studies had high risk of bias in blinding of participants and personnel, and blinding of outcome assessment; 76.9% had high risk of bias in incomplete outcome data; 15.4% of studies had high risk of bias in selective reporting; and 7.7% of studies had high risk of bias in other bias.

### 2.5. Statistical Analysis

All outcomes were continuous variables. Pooled analyses were completed using Review Manager 5.4 and Stata 14. Means and standard deviations of the raw data were to be collected if the included data units were consistent. If inconsistent, they were to be assessed using standardized mean differences. The effect sizes and confidence intervals of multiple studies combined were depicted by forest plots and tested for stability using sensitivity analysis. When the pooled statistics are as follows, we think of them as having low heterogeneity, moderate heterogeneity, and high heterogeneity: I^2^ ≤ 50%, 50–75%, and I^2^ > 75%. Begg’s test assessed publication bias. *p* < 0.05, with a confidence interval of 95%, was considered statistically significant.

## 3. Results

### 3.1. Review Characteristics

This study summarizes the 13 randomized controlled trial studies (Supplementary Materials S2). It includes first author, year, sample size, background, country, age, period, instruments for measuring GS and muscle mass, and exercise interventions. There was a total of 722 of these participants, with an age range of 63–92 years; the exercise programs include high, moderate, and low-intensity resistance training, balance training, kettlebell training, Nordic walking, and whole-body vibration training, and the duration of the interventions ranged from 8 to 24 weeks.

### 3.2. The Effect on Grip Strength

**Figure 3:**
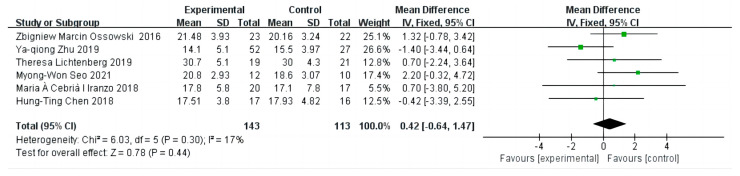
The forest maps of effect sizes of grip strength at baseline. The green squares in the middle of each horizontal line indicates a single study’s point estimate of the effect, and the size of the squares is proportional to the study’s weight in relation to the pooled estimate. The black diamond indicates the meta-analysis’s overall effect estimate, while the diameter of the diamond is the 95% CI around the pooled effect point estimate.

The combined results of the baseline period effect sizes are shown in Figure 3, with six [12,21,22,23,24,25] studies that chose GS to assess the effect of exercise in older adults with sarcopenia, including 256 individuals (EG = 143, CG = 113). There was no heterogeneity and no significant difference between the two groups at baseline (I^2^ = 17% < 50%, *p* = 0.44 > 0.05), so a fixed-effects model was chosen for the analysis, and the combined effect size at baseline was 0.42 (95% CI: −0.64 to 1.47).

**Figure 4:**
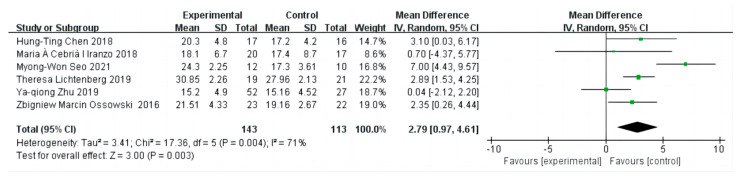
The forest plot of effect sizes of different exercise interventions compared to control on grip strength. The green squares in the middle of each horizontal line indicates a single study’s point estimate of the effect, and the size of the squares is proportional to the study’s weight in relation to the pooled estimate. The black diamond indicates the meta-analysis’s overall effect estimate, while the diameter of the diamond is the 95% CI around the pooled effect point estimate.

The forest plot in Figure 4 shows that the effect sizes of GS merging in patients with sarcopenia after the intervention was found to be highly heterogeneous by the heterogeneity test (I^2^ = 71%, *p* = 0.004 < 0.05), so a random-effects model was chosen for the meta-analysis. GS was chosen to assess the effect of exercise in elderly patients with sarcopenia, with six trials incorporating information about GS. Combining multiple exercise modalities (resistance training, kettlebell training, short-term Nordic walking, whole-body vibration therapy, and high-intensity resistance training), the final combined effect size was found to be 2.79 and was significant (95% CI: 0.97 to 4.61, Z = 3.00, *p* = 0.003 < 0.05). This indicates a substantial increase of 2.79 times in GS after the intervention compared to the control group. The effect of the intervention was significant and statistically significant.

### 3.3. Sensitivity Analysis and Publication Bias

Sensitivity analyses of the six current papers revealed that none of them would have had a significant impact on the results of the study (Supplementary Materials S3). This indicates that the results of this study are stable. To test the publication bias of the six pieces of literature in this study, it can be seen that the funnel plot is basically symmetrical (Supplementary Materials S3). At the same time, Begg’s test *p* = 0.707 > 0.05, so it can be determined that there is no publication bias in the literature of this study.

### 3.4. The Effect on Knee Extensor Strength

**Figure 5:**
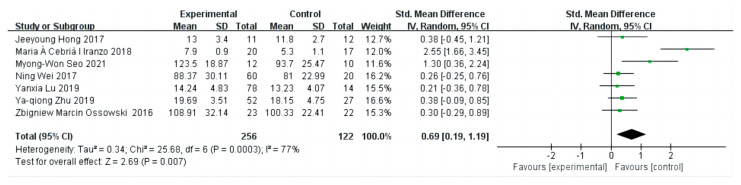
The forest plot of effect sizes of different exercise interventions compared to control on knee extensor strength. The green squares in the middle of each horizontal line indicates a single study’s point estimate of the effect, and the size of the squares is proportional to the study’s weight in relation to the pooled estimate. The black diamond indicates the meta-analysis’s overall effect estimate, while the diameter of the diamond is the 95% CI around the pooled effect point estimate.

In the forest plot of Figure 5, the effect sizes of the combined KES are shown and were found to be highly heterogeneous by the heterogeneity test (I^2^ = 77%, *p* = 0.0003 < 0.01), and were therefore analyzed using a random-effects model. The KES was chosen to assess the effect of exercise in elderly patients with sarcopenia, with seven trials incorporating information about the KES [21,22,23,25,26,27,28]. Combining multiple exercise modalities (combined resistance and balance training, resistance training, supervised resistance training, short-term Nordic walking, and whole-body vibration training) ultimately found an effect size of 0.69 after meta-merge and a significant effect size (95% CI: 0.19 to 1.19, Z = 2.69, *p* = 0.007 < 0.05). This indicates that the KES was significantly 0.69 times higher after the intervention than in the control group.

### 3.5. Sensitivity Analysis and Publication Bias

Sensitivity analyses of the seven current papers revealed that none of them would have had a significant impact on the results of the study (Supplementary Materials S3). To further test the publication bias of the existing seven pieces of literature, it can be seen that the funnel plot is basically symmetrical (Supplementary Materials S3). At the same time, the Egger’s test *p* = 0.133 > 0.05, so it can be determined that there is basically no publication bias in the literature of the current study.

### 3.6. The Effect on Appendicular Skeletal Muscle Index

**Figure 6:**
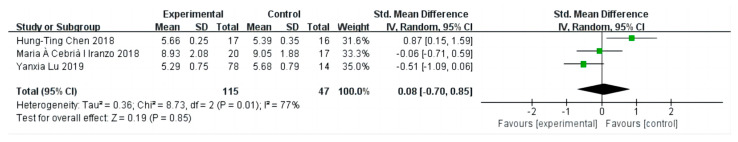
The forest plot of effect sizes of different exercise interventions compared to control on appendicular skeletal muscle index. The green squares in the middle of each horizontal line indicates a single study’s point estimate of the effect, and the size of the squares is proportional to the study’s weight in relation to the pooled estimate. The black diamond indicates the meta-analysis’s overall effect estimate, while the diameter of the diamond is the 95% CI around the pooled effect point estimate.

In the forest plot of Figure 6, the effect sizes of the combined ASMI are shown and were found to be highly heterogeneous by the heterogeneity test (I^2^ = 77%, *p* = 0.01 < 0.05), and were therefore analyzed using a random-effects model. The ASMI was chosen to assess the effect of exercise in elderly patients with sarcopenia, and three trials included information on the ASMI [12,25,28]. Combining multiple exercise modalities (combined resistance and balance, kettlebell, and resistance training) ultimately revealed a meta-merged ASMI effect size of 0.08. However, the effect size was insignificant (95% CI: −0.70 to 0.85, Z = 0.19, *p* = 0.85 > 0.05). This suggests that the multiple exercise modalities’ intervention effect on ASMI was insignificant and had no statistical significance compared with the control group.

### 3.7. Sensitivity Analysis and Publication Bias

Sensitivity analyses of the three current papers revealed that none of them would have had a significant impact on the results of the study (Supplementary Materials S3). To further test the publication bias of the existing three pieces of literature, it can be seen that the funnel plot is basically symmetrical (Supplementary Materials S3). At the same time, Begg’s test was also conducted to conclude that *p* = 0.296 > 0.05, so it can be determined that there may be no publication bias in the literature of the current study.

### 3.8. The Effect on Skeletal Muscle Index

**Figure 7:**
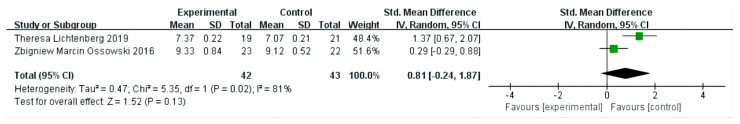
The forest plot of effect sizes of different exercise interventions compared to control on skeletal muscle index. The green squares in the middle of each horizontal line indicates a single study’s point estimate of the effect, and the size of the squares is proportional to the study’s weight in relation to the pooled estimate. The black diamond indicates the meta-analysis’s overall effect estimate, while the diameter of the diamond is the 95% CI around the pooled effect point estimate.

In the forest plot of Figure 7, the combined SMI’s effect sizes are shown and analyzed using a random-effects model after a test for heterogeneity revealed a high degree of heterogeneity (I^2^ = 81%, *p* = 0.02 < 0.05). SMI was chosen to assess the effect of exercise in elderly patients with sarcopenia, and two trials included information on the SMI [22,24]. Combining multiple exercise modalities (short-term Nordic walking and high-intensity resistance training) ultimately revealed a meta-merged effect size of 0.81 for SMI. However, the effect size was insignificant (95% CI: −0.24 to 1.87, Z = 1.52, *p* = 0.13 > 0.05). This suggests that the intervention effect of multiple exercise modalities on SMI was not significant and statistically significant compared to the control group.

### 3.9. Sensitivity Analysis and Publication Bias

Sensitivity analyses of the two current papers revealed that none of them would have had a significant impact on the results of the study (Supplementary Materials S3). To further test the publication bias of the existing two pieces of literature, it can be seen that the funnel plot is basically symmetrical. However, due to the limited number of included studies, there may be some bias in their test results. Therefore, we found the difference between the before and after results by clipping and patching (Supplementary Materials S3). After three iterations, one document was finally virtualized, there was no publication bias in the total of three papers after clipping and patching (*p* = 0.618 > 0.05), and the combined effect size was 0.299 (95% CI: −0.876 to 1.475). Finally, we compared the results before clipping and after clipping, and there were inconsistencies in the results; it can be concluded that the SMI results of the existing meta-analysis are unstable, and the results of the meta-analysis will be further varied if there are new studies in the future.

## 4. Discussion

In this meta-analysis study, 13 randomized controlled trials were examined to compare the effects of different exercise interventions on GS, KES, ASMI, and SMI in older adults with sarcopenia. The findings revealed that resistance training, kettlebell training, short-term Nordic walking, whole-body vibration therapy, and high-intensity resistance training were all effective in improving GS. Furthermore, interventions such as mixed training, supervised resistance training, short-term Nordic walking, and whole-body vibration therapy were shown to significantly increase KES. However, there were no significant differences observed in ASMI and SMI among the various interventions.

In the updated definition proposed by the European Working Group on Sarcopenia in Older People 2 (EWGSOP2), GS is used as a measure of muscle strength [29,30] and is one of the most commonly used measurements in daily life. Almost all of the GS outcome indicators in this review had an intervention duration of more than 12 weeks, with only one having a duration of 8 weeks. For the previous studies with intervention periods of less than 12 weeks, we found that only 8 weeks of GS training, including static stretching exercises for the forearm, wrist, and fingers, in older women significantly increased maximal GS in the intervention group compared to the control group [31].

On the other hand, in the study where the intervention lasted for 36 months, the older adults did not find any favorable effect on GS [32]. This finding may be attributed to the control condition requiring the completion of home exercises. Additionally, the present study’s results found that using either a combination of exercises or a single exercise within a practical intervention time frame could benefit GS in older adults with sarcopenia. Shiotsu Y studied GS in older adults through aerobic combined resistance training and found that significant differences were observed in participants’ GS regardless of the sequencing of aerobic and resistance training [33]. In contrast, Song MS intervened in older women over 65 years of age through a single Nordic walking intervention and found that, after 12 weeks of isometric exercise with a long pole in hand, not only did the body composition improve but there was also an increase in strength in terms of GS [34]. Therefore, when improving GS in older adults with sarcopenia, it is essential to consider the intervention condition as fully as possible.

The results of our meta-analysis suggest that resistance training, whole-body vibration training, Nordic walking training, and resistance combined with balance training are effective in improving lower extremity muscle strength in older adults with sarcopenia. Zhu YQ’s findings are consistent with the present review, and explored the effects of tai chi and whole-body vibration training on older patients with sarcopenia [24]. The study found that, after a long period of resistance training and whole-body vibration exercise, the patients showed significant improvement in terms of lower limb muscle strength. It is worth noting that, in the tai chi exercise program, the increase in lower limb strength is directly related to the characteristics of this exercise program, which is a dynamic movement premised on lowering the center of gravity. The quadriceps muscles play a major role in this process. Previous systematic reviews and meta-analyses have also agreed that training/therapy with vibratory modalities, either localized vibratory therapy or whole-body vibration, enhances KES in older adults [35,36]. However, in a study of 80 older adults with age-related muscle loss, Wei N compared the effects of different combinations of vibration frequencies (20 Hz, 40 Hz, 60 Hz) on KES. The results showed that the best results were achieved when the vibration frequency was 40 Hz [27]. In terms of Nordic walking training, Lee HS investigated the effects of Nordic walking on frail people over 70 years of age. The results showed that lower limb muscle strength showed statistically significant improvement in frail people after Nordic walking training [37]. For resistance combined with balance training, in a randomized controlled trial of sarcopenia patients aged 80–99 years, Liang Y demonstrated that resistance training combined with balance training significantly improved lower limb function compared to resistance training alone [38].

The present meta-analysis found that no substantial improvement in ASMI and SMI could be achieved with any exercise. This may be due to inadequate nutritional intake and increased protein catabolism, and decreased anabolism in older adults with advancing age [39]. Low ASMI and SMI are considered important indicators of decreased muscle mass [40,41]. While muscle mass loss is a normal physiological phenomenon during aging, changes in muscle fiber structure within the body, improved neuronal activity, and increased muscle strength can be detected through training without changes in muscle mass [42]. As Santos L reported, resistance training increased gait speed and muscle strength in older women but was not associated with muscle mass or fat mass [43].

Similarly, in a chair-based exercise program, chair-based resistance exercises with elastic bands did not significantly increase muscle mass in older adults over 80 but improved strength and balance [44]. Typically, improvement in sarcopenia is more commonly achieved in clinical settings using a combination of nutritional interventions and exercise programs [45]. This conclusion has previously been shown to increase effectiveness, and, with adequate dietary intake and appropriate exercise regimens, muscle strength and mass will delay decline. However, some older adults are still unable or sometimes only able to participate in activity programs for a short period of time. Research needs to be more comprehensive from a nutritional intervention perspective, primarily focusing on muscle mass or muscle strength loss in sarcopenia patients through nutritional intervention alone [46]. In the only studies available so far, it has been found that leucine plays a vital role in muscle growth as it is the most effective amino acid for stimulating muscle protein synthesis [47]. Accordingly, it was shown that supplementation that was protein-rich (including leucine) and contained vitamin D increased muscle mass in a previous randomized, double-blind, placebo-controlled trial [48]. This study was conducted in an adult population based in late middle age to emphasize that early nutritional intervention is used to counteract sarcopenia itself.

## 5. Strengths and Limitations

This paper has specific strengths as well: firstly, all 13 articles included in this meta-analysis were randomized controlled trials, which can lead to a more objective assessment of the outcome indicators. Secondly, the inclusion criteria for this meta-analysis were quite stringent, focusing solely on elderly patients with sarcopenia.

This meta-analysis explored the effects of exercise on GS, KES, ASMI, and SMI in older adults with sarcopenia. However, our findings need to be interpreted with caution due to potential limitations. Firstly, there was a large heterogeneity in some of the results of this study. This may be due to the diversity of exercise modalities, gender inconsistency, and age differences. Secondly, differences in diagnostic criteria for sarcopenia may have led to inconsistencies in the methods and instruments used to measure muscle-related functions. This affected inter-study comparisons to some extent. Finally, due to the limited number of studies included in this meta-analysis, subgroup analyses of age groups were not performed in this paper. The age range of the included participants varied widely, ranging from 63 to 92 years. Future studies should include more high-quality articles with subgroups of age groups, diagnostic criteria, and measurement tools. This approach will enable a more comprehensive evaluation of the impacts of exercise interventions on muscle strength and mass in older adults with sarcopenia.

## 6. Conclusions

For elderly patients with sarcopenia, the results of this study provide relatively reliable data for clinical stakeholders. Compared to ASMI and SMI, exercise intervention can improve muscle strength (GS and KES), especially through resistance training. Secondly, combined training may also be an effective way to prevent and treat sarcopenia in older adults. In addition to the existing literature on intervention modalities, future studies could consider and evaluate exercise frequency, duration, intensity, and age group. Additionally, we also suggest that a new consensus on sarcopenia should address some standardization; even though this may be difficult for clinical use, the results can be compared between research fields and researchers using standardized assessment methods.

## Data Availability

The original contributions presented in the study are included in the article. Further inquiries can be directed to the corresponding author.

## Supplementary Materials

The following supporting information can be downloaded at: https://www.mdpi.com/article/10.3390/diseases12040071/s1, Supplementary Materials S1: search strategy; Supplementary Materials S2: Demographic information; Supplementary Materials S3: sensitivity analysis plot; funnel plot.
