# Supplementary material for: Effect of Different Exercise Interventions on Grip Strength, Knee Extensor Strength, Appendicular Skeletal Muscle Index, and Skeletal Muscle Index Strength in Patients with Sarcopenia: A Meta-Analysis of Randomized Controlled Trials
Source: Diseases. 2024 Apr 2;12(4):71. doi: 10.3390/diseases12040071 (PMC11049519; doi:10.3390/diseases12040071)
Supplement: Supplementary file 1 [file diseases-12-00071-s001.zip › Supplementary Materials S1.pdf]

Supplementary Materials S1: search strategy

Effect of different exercise interventions on grip strength, knee extensor strength, appendicular skeletal muscle index and skeletal muscle index strength in patients with sarcopenia: a meta-analysis of randomized controlled trials

PubMed

Layer	Search Terms	Results
#1	"Aged"[MeSH Terms] OR "aged, 80 and over"[MeSH Terms] OR "Frail Elderly"[MeSH Terms] OR "Geriatrics"[MeSH Terms] OR "Geriatric Psychiatry"[MeSH Terms] OR "Geriatric Nursing"[MeSH Terms] OR "Dental Care for Aged"[MeSH Terms] OR "Health Services for the Aged"[MeSH Terms] OR "elder*"[Title/Abstract] OR "eldest"[Title/Abstract] OR "frail*"[Title/Abstract] OR "geriatri*"[Title/Abstract] OR "old age*"[Title/Abstract] OR "oldest old*"[Title/Abstract] OR "senior*"[Title/Abstract] OR "senium"[Title/Abstract] OR "very old*"[Title/Abstract] OR "septuagenarian*"[Title/Abstract] OR "octagenarian*"[Title/Abstract] OR "octogenarian*"[Title/Abstract] OR "nonagenarian*"[Title/Abstract] OR "centarian*"[Title/Abstract] OR "centenarian*"[Title/Abstract] OR "supercentenarian*"[Title/Abstract] OR "older people"[Title/Abstract] OR "older subject*"[Title/Abstract] OR "older patient*"[Title/Abstract] OR "older age*"[Title/Abstract] OR "older adult*"[Title/Abstract] OR "older man"[Title/Abstract] OR "older men"[Title/Abstract] OR "older male"[Title/Abstract] OR "older woman"[Title/Abstract] OR "older women"[Title/Abstract] OR "older female"[Title/Abstract] OR "older population*"[Title/Abstract] OR "older person*"[Title/Abstract]	3683951

**#2**	"Exercise"[MeSH Terms] OR ("physical activity"[Title/Abstract] OR "activities physical"[Title/Abstract] OR "activity physical"[Title/Abstract] OR "physical activities"[Title/Abstract] OR "exercise physical"[Title/Abstract] OR "exercises physical"[Title/Abstract] OR "physical exercise"[Title/Abstract] OR "physical exercises"[Title/Abstract] OR "exercise isometric"[Title/Abstract] OR "exercises isometric"[Title/Abstract] OR "isometric exercises"[Title/Abstract] OR "isometric exercise"[Title/Abstract] OR "exercise aerobic"[Title/Abstract] OR "aerobic exercise"[Title/Abstract] OR "aerobic exercises"[Title/Abstract] OR "exercises aerobic"[Title/Abstract] OR "exercise training"[Title/Abstract] OR "exercise trainings"[Title/Abstract] OR "training exercise"[Title/Abstract] OR (("education"[MeSH Subheading] OR "education"[All Fields] OR "Training"[All Fields] OR "education"[MeSH Terms] OR "train"[All Fields] OR "trains"[All Fields] OR "trained"[All Fields] OR "trainings"[All Fields] OR "Trainings"[All Fields] OR "trains"[All Fields]) OR "Exercise"[Title/Abstract]))	2805530
**#3**	"Sarcopenia"[MeSH Terms] OR "Sarcopenia"[Title/Abstract]	15628
**#4**	"randomized controlled trial"[Title/Abstract] OR "cluster randomized controlled trial"[Title/Abstract] OR "randomised controlled trial"[Title/Abstract] OR "cluster randomised controlled trial"[Title/Abstract]	134871
**#5**	#1 AND #2 AND #3 AND #4	152

Web of science

Layer	Search Terms	Results
**#1**	TS=(Aged OR aged, 80 and over OR Frail Elderly OR Geriatrics OR Geriatric Psychiatry OR Geriatric Nursing OR Dental Care for Aged OR Health Services for the Aged OR elder* OR eldest OR frail* OR geriatri* OR old age* OR oldest old* OR senior* OR senium OR very old* OR septuagenarian* OR octagenarian* OR octogenarian* OR nonagenarian* OR centarian* OR centenarian* OR supercentenarian* OR older people OR older subject* OR older patient* OR older age* OR older adult* OR older man OR older men OR older male OR older woman OR older women OR older female OR older population* OR older person*)	11327887
**#2**	TS=(movement behavio* OR 24-h* OR Exercise OR physical activity OR activities physical OR activity physical OR physical activities OR exercise physical OR exercises physical OR physical exercise OR physical exercises OR exercise isometric OR exercises isometric OR isometric exercises OR isometric exercise OR exercise aerobic OR	1462437

	aerobic exercise OR aerobic exercises OR exercises aerobic OR exercise training OR exercise trainings OR training exercise)	
**#3**	TS=(Sarcopenia)	25086
**#4**	TS=(randomized controlled trial OR cluster randomized controlled trial OR randomised controlled trial OR cluster randomised controlled trial)	707831
**#5**	#1 AND #2 AND #3 AND #4	772

The Cochrane Library

Layer	Search Terms	Results
**#1**	MeSH descriptor: [Aged] explode all trees	221613
**#2**	MeSH descriptor: [Aged, 80 and over] explode all trees	56156
**#3**	MeSH descriptor: [Frail Elderly] explode all trees	804
**#4**	MeSH descriptor: [Geriatrics] explode all trees	214
**#5**	MeSH descriptor: [Geriatric Psychiatry] explode all trees	41
**#6**	MeSH descriptor: [Geriatric Nursing] explode all trees	180
**#7**	MeSH descriptor: [Dental Care for Aged] explode all trees	66
**#8**	MeSH descriptor: [Health Services for the Aged] explode all trees	462
**#9**	(elder*):ab,ti,kw OR (eldest):ab,ti,kw OR (frail*):ab,ti,kw OR (geriatri*):ab,ti,kw OR (old age*):ab,ti,kw OR (oldest old*):ab,ti,kw OR (senior*):ab,ti,kw OR (senium):ab,ti,kw OR (very old*):ab,ti,kw OR (septuagenarian*):ab,ti,kw OR (octagenarian*):ab,ti,kw OR (octogenarian*):ab,ti,kw OR (nonagenarian*):ab,ti,kw OR (centarian*):ab,ti,kw OR (centenarian*):ab,ti,kw OR (supercentenarian*):ab,ti,kw OR (older people):ab,ti,kw OR (older subject*):ab,ti,kw OR (older patient*):ab,ti,kw OR (older age*):ab,ti,kw OR (older adult*):ab,ti,kw OR (older man):ab,ti,kw OR (older men):ab,ti,kw OR (older male):ab,ti,kw OR (older woman):ab,ti,kw OR (older women):ab,ti,kw OR (older female):ab,ti,kw OR (older population*):ab,ti,kw OR (older person*):ab,ti,kw	159832
**#10**	#1 OR #2 OR #3 OR #4 OR #5 OR #6 OR #7 OR #8 OR #9	349867

**#11**	(Exercise):ab,ti,kw OR (physical activity):ab,ti,kw OR (activities physical):ab,ti,kw OR (activity physical):ab,ti,kw OR (physical activities):ab,ti,kw OR (exercise physical):ab,ti,kw OR (exercises physical):ab,ti,kw OR (physical exercise):ab,ti,kw OR (physical exercises):ab,ti,kw OR (exercise isometric):ab,ti,kw OR (exercises isometric):ab,ti,kw OR (isometric exercises):ab,ti,kw OR (isometric exercise):ab,ti,kw OR (exercise aerobic):ab,ti,kw OR (aerobic exercise):ab,ti,kw OR (aerobic exercises):ab,ti,kw OR (exercises aerobic):ab,ti,kw OR (exercise training):ab,ti,kw OR (exercise trainings):ab,ti,kw OR (training exercise):ab,ti,kw	141873
**#12**	(Sarcopenia):ab,ti,kw	1979
**#13**	(randomized controlled trial):ab,ti,kw OR (cluster randomized controlled trial):ab,ti,kw OR (randomised controlled trial):ab,ti,kw OR (cluster randomised controlled trial):ab,ti,kw	676120
**#14**	#10 AND #11 AND #12 AND #13	458

Embase

Layer	Search Terms	Results
**#1**	aged	5510390
**#2**	aged, AND 80 AND over	69487
**#3**	frail AND elderly	21819
**#4**	geriatrics	176223
**#5**	geriatric AND psychiatry	33575
**#6**	geriatric AND nursing	43487
**#7**	dental AND care AND for AND aged	21013
**#8**	health AND services AND for AND the AND aged	164456
**#9**	#1 OR #2 OR #3 OR #4 OR #5 OR #6 OR #7 OR #8	5614130
**#10**	'elder*':ab,ti OR 'eldest':ab,ti OR 'frail*':ab,ti OR 'geriatri*':ab,ti OR 'old age*':ab,ti OR 'oldest old*':ab,ti OR 'senior*':ab,ti OR 'senium':ab,ti OR 'very old*':ab,ti OR 'septuagenarian*':ab,ti OR 'octagenarian*':ab,ti OR 'octogenarian*':ab,ti OR 'nonagenarian*':ab,ti OR 'centarian*':ab,ti OR 'centenarian*':ab,ti OR 'supercentenarian*':ab,ti OR 'older people':ab,ti OR 'older subject*':ab,ti OR 'older patient*':ab,ti OR 'older age*':ab,ti OR 'older adult*':ab,ti OR 'older man':ab,ti OR 'older men':ab,ti OR 'older male':ab,ti OR 'older woman':ab,ti OR 'older women':ab,ti OR 'older female':ab,ti OR 'older population*':ab,ti OR 'older person*':ab,ti	901960
**#11**	#9 OR #10	5866423
**#12**	exercise	665606

**#13**	'physical activity':ab,ti OR 'activities physical':ab,ti OR 'activity physical':ab,ti OR 'physical activities':ab,ti OR 'exercise physical':ab,ti OR 'exercises physical':ab,ti OR 'physical exercise':ab,ti OR 'physical exercises':ab,ti OR 'exercise isometric':ab,ti OR 'exercises isometric':ab,ti OR 'isometric exercises':ab,ti OR 'isometric exercise':ab,ti OR 'exercise aerobic':ab,ti OR 'aerobic exercise':ab,ti OR 'aerobic exercises':ab,ti OR 'exercises aerobic':ab,ti OR 'exercise training':ab,ti OR 'exercise trainings':ab,ti OR 'training exercise':ab,ti	250451
**#14**	#12 OR #13	766343
**#15**	sarcopenia	26867
**#16**	randomized AND controlled AND trial	1073857
**#17**	'cluster randomized controlled trial':ab,ti OR 'randomised controlled trial':ab,ti OR 'cluster randomised controlled trial':ab,ti	43709
**#18**	#16 OR #17	1079312
**#19**	#11 AND #14 AND #15 AND #18	781
