# Supplementary material for: Effect of Different Exercise Interventions on Grip Strength, Knee Extensor Strength, Appendicular Skeletal Muscle Index, and Skeletal Muscle Index Strength in Patients with Sarcopenia: A Meta-Analysis of Randomized Controlled Trials
Source: Diseases. 2024 Apr 2;12(4):71. doi: 10.3390/diseases12040071 (PMC11049519; doi:10.3390/diseases12040071)
Supplement: Supplementary file 1 [file diseases-12-00071-s001.zip › Supplementary Materials S3.pdf]

Supplementary Materials S3: sensitivity analysis plot; funnel plot.

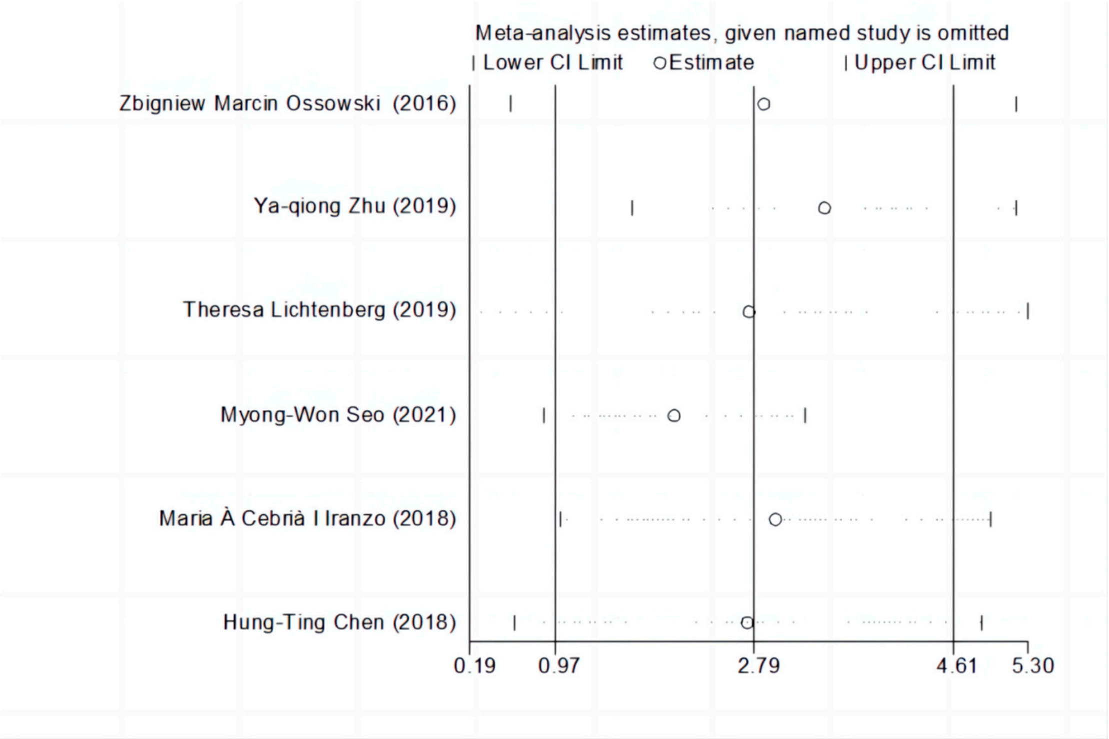

Figure 1. The sensitivity analysis results of grip strength.

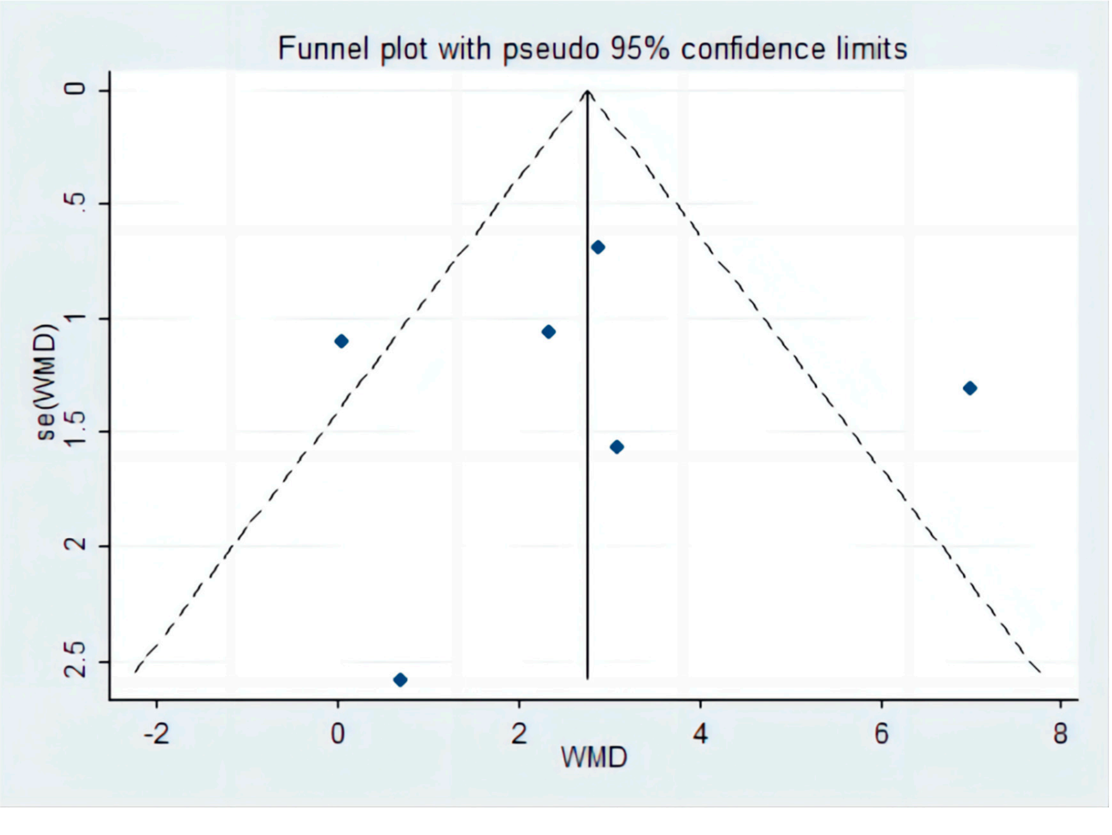

Figure 2. The funnel plot results of grip strength.

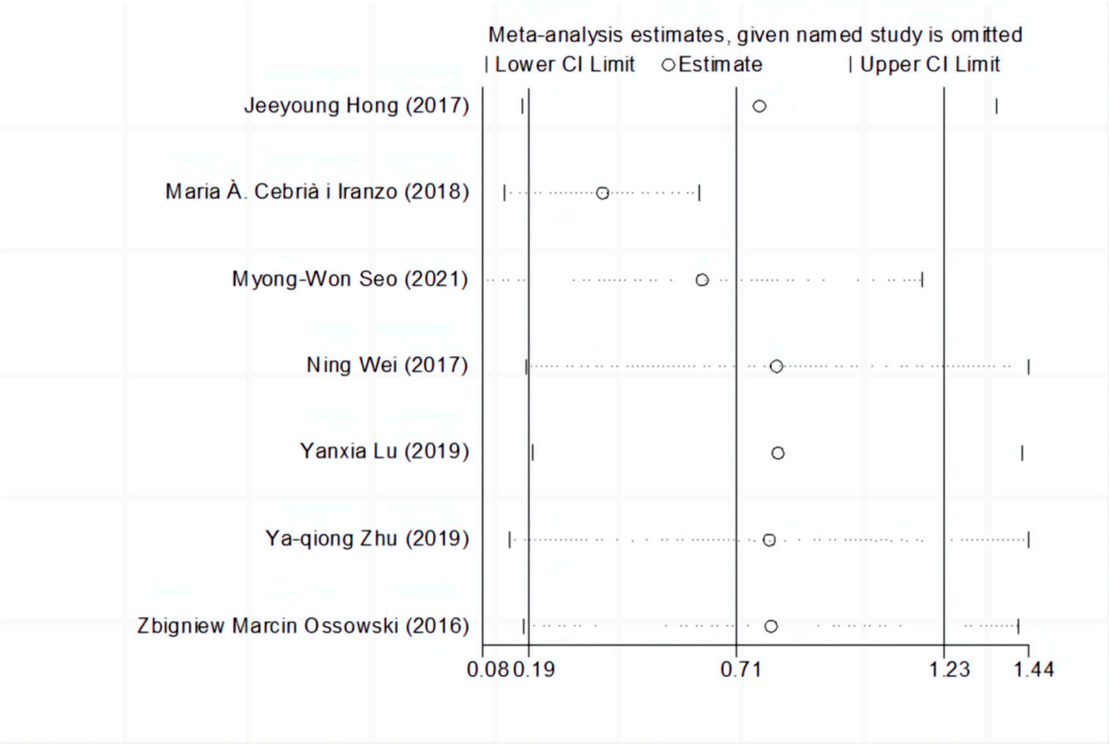

Figure 3. The sensitivity analysis results of knee extensor strength.

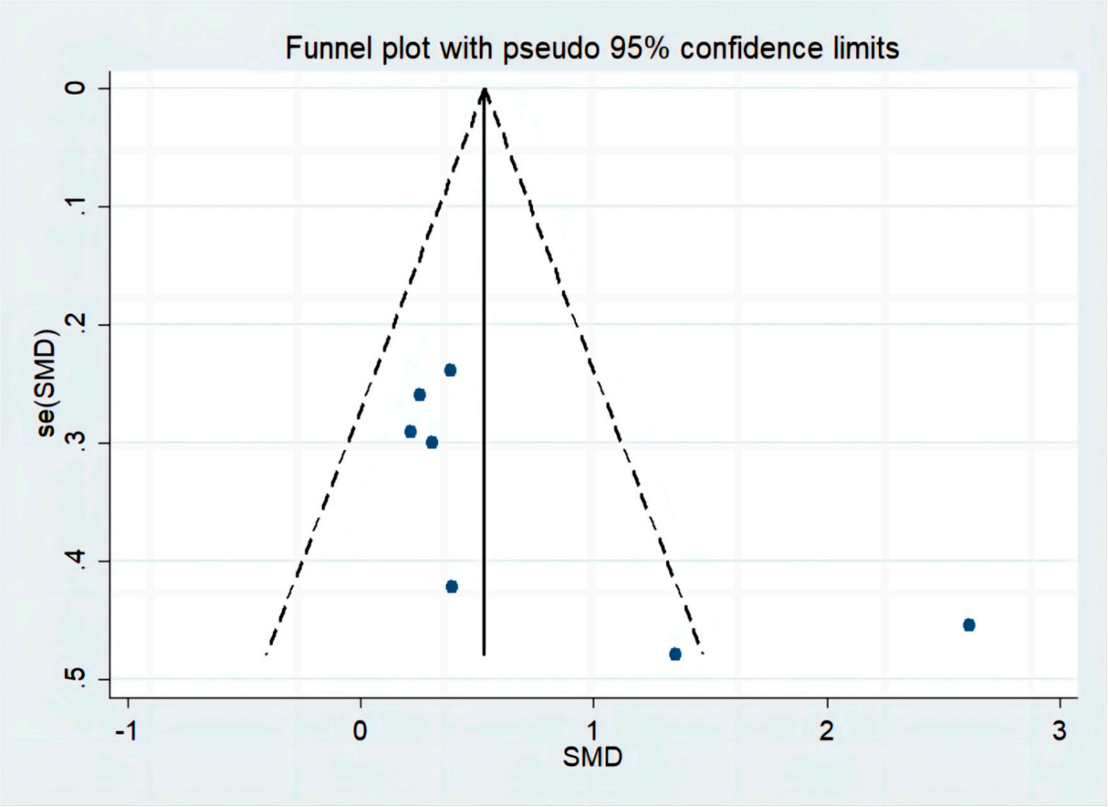

Figure 4. The funnel plot results of knee extensor strength.

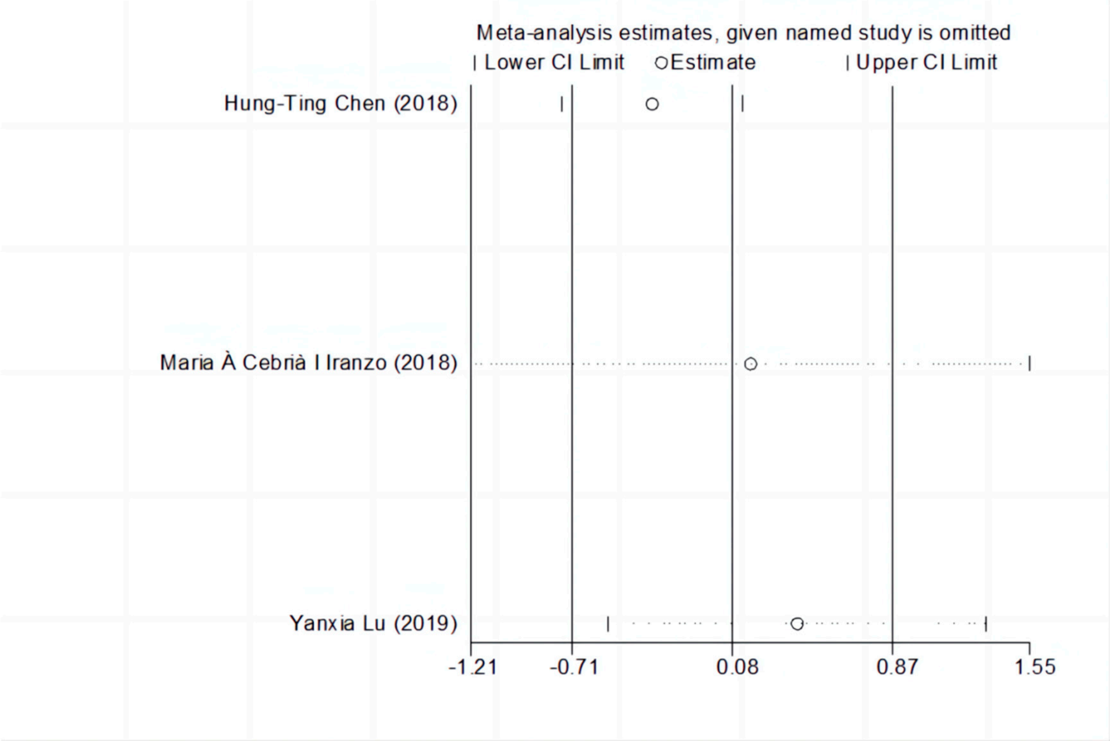

Figure 5. The sensitivity analysis results of appendicular skeletal muscle index.

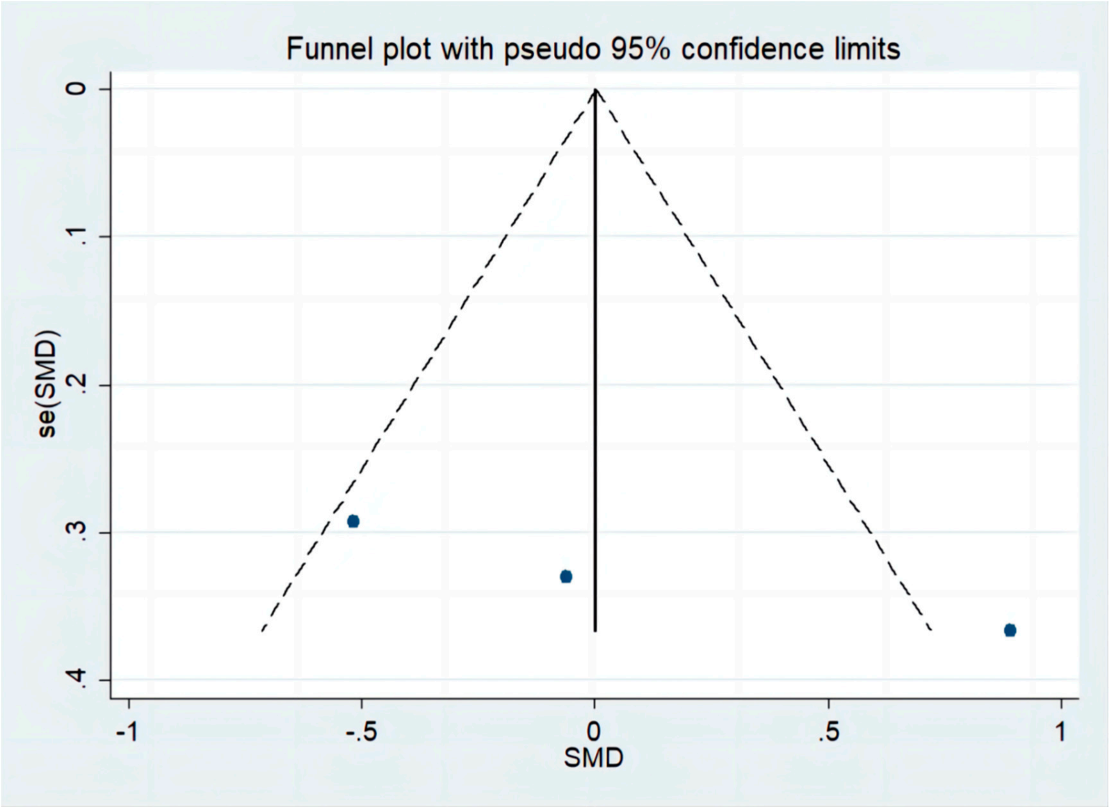

Figure 6. The funnel plot results of appendicular skeletal muscle index.

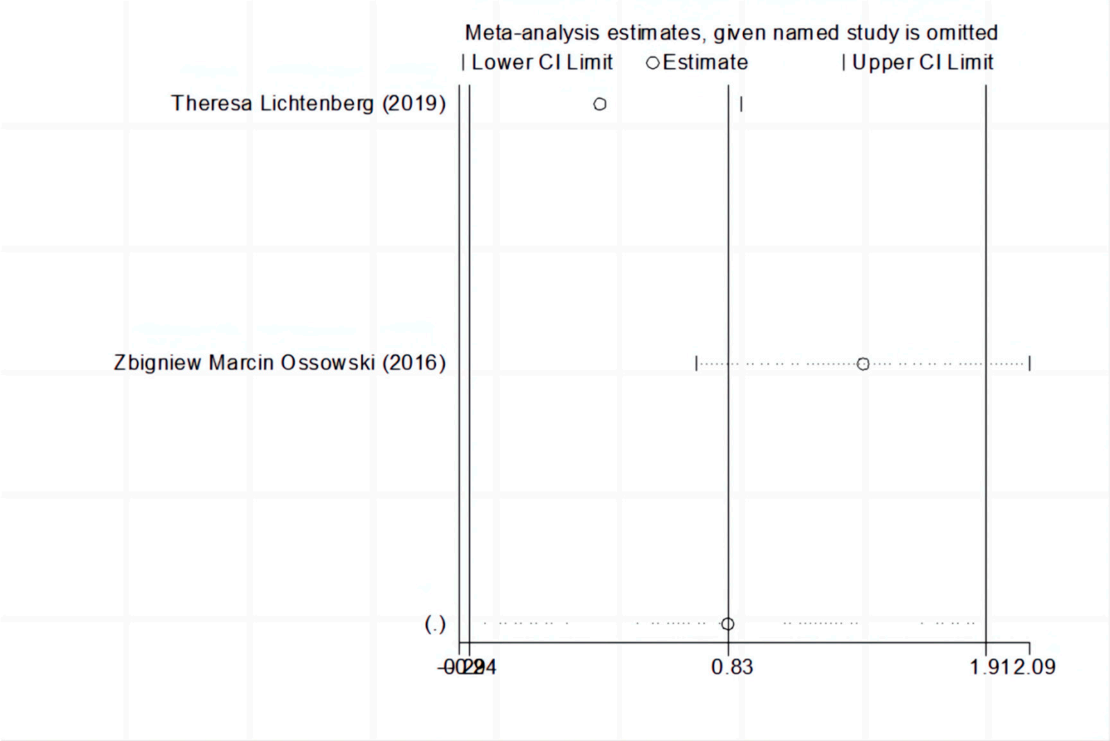

Figure 7. The sensitivity analysis results of appendicular skeletal muscle index.

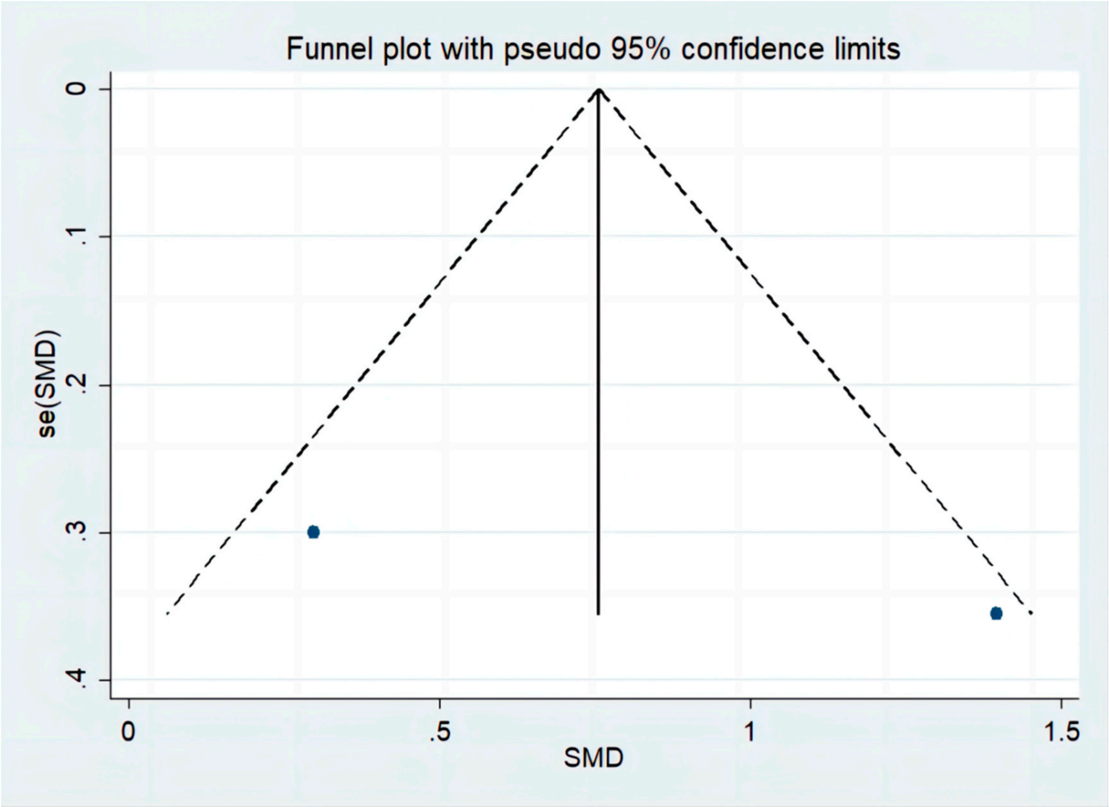

Figure 8. The funnel plot results of skeletal muscle index.

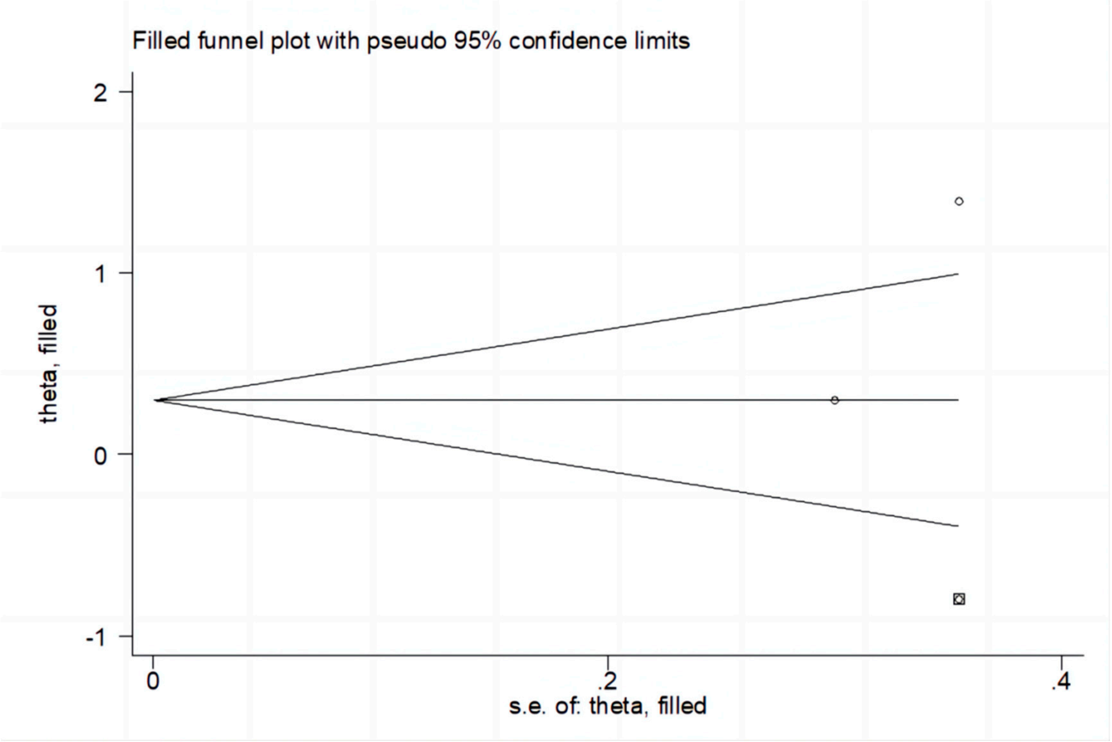

Figure 9. The funnel plot after trimmed.
